# Ecological and Evolutionary responses to Antibiotic Treatment in the Human Gut Microbiota

**DOI:** 10.1093/femsre/fuab018

**Published:** 2021-04-06

**Authors:** Joseph Hugh Pennycook, Pauline Deirdre Scanlan

**Affiliations:** APC Microbiome Ireland, Biosciences Institute, University College Cork, College Road, Cork, T12 YT20, Ireland; School of Mirobiology, Food Science & Technology Building, University College Cork, College Road, Cork, T12 K8AF, Ireland; APC Microbiome Ireland, Biosciences Institute, University College Cork, College Road, Cork, T12 YT20, Ireland; School of Mirobiology, Food Science & Technology Building, University College Cork, College Road, Cork, T12 K8AF, Ireland

**Keywords:** antibiotics, gut microbiota, diversity, ecology, evolution

## Abstract

The potential for antibiotics to affect the ecology and evolution of the human gut microbiota is well recognised and has wide-ranging implications for host health. Here, we review the findings of key studies that surveyed the human gut microbiota during antibiotic treatment. We find several broad patterns including the loss of diversity, disturbance of community composition, suppression of bacteria in the Actinobacteria phylum, amplification of bacteria in the Bacteroidetes phylum, and promotion of antibiotic resistance. Such changes to the microbiota were often, but not always, recovered following the end of treatment. However, many studies reported unique and/or contradictory results, which highlights our inability to meaningfully predict or explain the effects of antibiotic treatment on the human gut microbiome. This problem arises from variation between existing studies in three major categories: differences in dose, class and combinations of antibiotic treatments used; differences in demographics, lifestyles, and locations of subjects; and differences in measurements, analyses and reporting styles used by researchers. To overcome this, we suggest two integrated approaches: (i) a top-down approach focused on building predictive models through large sample sizes, deep metagenomic sequencing, and effective collaboration; and (ii) a bottom-up reductionist approach focused on testing hypotheses using model systems.

## Teaser

Towards understanding and predicting the ecological and evolutionary responses to antibiotic treatment in the human gut microbiota.

## INTRODUCTION

Antibiotics—chemicals that kill or inhibit the growth of bacteria—play a central role in human medicine. They have been used in some form for at least 1,500 years (Bassett *et al*. 1980) and produced industrially since the end of the Second World War (Aminov 2010; Gould 2016), hugely improving the treatment efficacy of many bacterial infections such as tuberculosis (Blower, Small and Hopewell 1996), typhoid (Bavdekar *et al*. 1991) and syphilis (Dayan and Ooi 2005). About 34.8 billion defined daily doses of antibiotics were consumed worldwide in 2015 (Klein *et al*. 2018), and as of 2019, 38 antibiotic treatments were included on the World Health Organisation's Model List of Essential Medicines (World Health 2019).

Over the same time period, there has been increasing recognition among microbiologists that not all bacteria living in and on our bodies are harmful. Diverse communities of microorganisms, predominantly bacteria, colonise the skin, throat and vagina of healthy humans (Huttenhower *et al*. 2012). However, the vast majority of microbes associated with the human body reside in the intestinal tract, and in particular the large intestine, where more than 100 billion cells per gram content can be found (Walter and Ley 2011). This community—the gut microbiota (Marchesi and Ravel 2015)—has numerous impacts on the health of its host, including the transformation of metabolites into energy sources usable by the colonic epithelium cells (Duncan *et al*. 2007), calibration of the developing immune system (Thaiss *et al*. 2016), and production of neurotransmitters that impact mood and behaviour (Sampson and Mazmanian 2015). These combined effects are so great that the entire habitat—the gut microbiome (Marchesi and Ravel 2015)—is considered by some to be a ‘forgotten organ’ of the human body (O'Hara and Shanahan 2006), and has attracted much research attention in recent years.

Given that antibiotics are specifically employed to disrupt bacterial populations, it is no surprise that antibiotic treatment can have collateral effects on the gut microbiota. Probably the first reports of such consequences were found in cases of *Clostridioides difficile* infections associated with antibiotic treatment (Bartlett *et al*. 1978). In this condition, known as *Clostridioides difficile* associated diarrhoea (CDAD), the microbiota is disrupted by antibiotics (Young and Schmidt 2004), allowing the often-resistant *C. difficile* to bloom and produce large volumes of toxins that result in severe, chronic diarrhoea (Stanley *et al*. 2013). However, antibiotics are also implicated in far more subtle gut microbiota-mediated impacts on health such as asthma and dermatitis, especially during early life (Willing, Russell and Finlay 2011). It has been hypothesised that reduced exposure to microbes during childhood is detrimental to immune development, and partially responsible for the rise in allergic disorders over the last few decades (Noverr and Huffnagle 2005).

It must be remembered, however, that the gut microbiome is not simply a ‘virtual organ’ with impacts on human health, but a complex biological community in its own right. Over 1000 distinct species have been isolated from the human gut environment to date, and many more remain unidentified (Rajilić-Stojanović and de Vos 2014). Neither are the bacteria that comprise the gut microbiota functionally equivalent. They play a diverse range of roles within this complex ecosystem; for example, *Ruminococcus bromii* acts as a keystone degrader of diet-derived resistant starches (Ze *et al*. 2012), *Akkermansia muciniphila* specialises in digesting complex molecules secreted by the epithelial lining (Belzer and de Vos 2012), and *Blautia hydrogenotrophica* can generate energy and biomass from hydrogen and carbon dioxide alone (Rey *et al*. 2010). Antibiotic treatment poses a radical change to the environment of these organisms, in terms of both their immediate chemical surroundings and the competitive or beneficial relationships that they have with other species. Therefore, any effects of antibiotic treatment on human health are potentially mediated by the effects of treatment on the ecology and evolution of the gut microbiota.

Many studies have contributed to our current understanding of how antibiotics affect the ecology and evolution of the gut microbiota, some using animal models or *in vitro* experiments, but most by surveying changes in the microbiota of human subjects as they undergo antibiotic treatment. In this review we focus on the latter group to summarise and discuss what is currently known about the ecological and evolutionary effects of antibiotic treatment on the gut microbiota. The primary findings of key studies in this area are summarised in Tables 1, 2 and Table S1 (Supporting Information), and include a number that were previously examined elsewhere (Ferrer *et al*. 2017). Although these studies were distributed across multiple continents, the majority were conducted in Western countries, and collectively surveyed hundreds of subjects as they were exposed to various antibiotic treatments. Some followed medical patients as they received necessary treatment for disease, producing clinically relevant results and reducing unnecessary exposure, while others administered antibiotics to healthy volunteers, achieving greater control and uniformity in their experiments (Table S1, Supporting Information). Most took a longitudinal approach, determining the effects of the antibiotics by comparing samples taken during and after treatment to those taken before exposure in the same subjects (De La Cochetière *et al*. 2005; Dethlefsen *et al*. 2008; Dethlefsen and Relman 2011; Bajaj *et al*. 2013; Pérez-Cobas *et al*. 2013b; Ladirat *et al*. 2014; Panda *et al*. 2014; Heinsen *et al*. 2015; Willmann *et al*. 2015; MacPherson *et al*. 2018, Suez *et al*. 2018), but a few took a cross-sectional approach and compared treated subjects only to untreated controls (O'Sullivan *et al*. 2013; Abeles *et al*. 2015; Wipperman *et al*. 2017), and several studies combined the two methods (Jernberg *et al*. 2007; Jakobsson *et al*. 2010; Morotomi *et al*. 2011; Arat *et al*. 2015; Rashid *et al*. 2015; Stewardson *et al*. 2015; Zaura *et al*. 2015; Raymond *et al*. 2016; Dubinsky *et al*. 2020). All reviewed studies surveyed the gut microbiota *via* faecal samples from their subjects, but these samples were analysed in various ways, including multiple different sequencing-based techniques, several molecular fingerprinting methods, or *in vitro* culture analyses (Table S1, Supporting Information), while a few studies took auxiliary measurements such as testing for metabolites in the serum and urine of their subjects (Bajaj *et al*. 2013), or direct sampling of the colonic mucosal microbiota (Heinsen *et al*. 2015; Suez *et al*. 2018). Despite the large variation in the methods and results of these studies, it is still possible to draw from them some broad conclusions about how the gut microbiota responds to antibiotic treatment. In addition, we highlight some key problems that have impeded progress in this field, and with those problems in mind suggest a set of guidelines for future research. In time, this approach should create a clear foundation of knowledge which can then be used to understand how antibiotic mediated effects on the gut microbiota are translated into consequences for human health.

**Table 1.** Results of studies that address the effects of antibiotic treatment on the human gut microbiota.

		Short Term Effects^b^							Long Term Effects^c^						
		Div^d^	Com^e^	Taxa^f^				ABR^g^	Div^d^	Comp^c^	Taxa^f^				ABR^g^
Reference	Treatment Mechanism^a^			ACT	BAC	FIR	PRO				ACT	BAC	FIR	PRO	
(De La Cochetière *et al*. 2005)	Cell Wall Synthesis Inhibitor		D	—	—	↑(2u)	↑(1u)			∼D	—	—	—	—	
(Jernberg *et al*. 2007)	Protein Synthesis Inhibitor	↓*	D*					↑	↓^h^	D^h^					↑
(Dethlefsen *et al*. 2008)	DNA Synthesis Inhibitor	↓*	D	|*(8u)	|*(72u)	|*(335u)	|*(8u)		—	—	—	|*(4u)	|*(39u)	|*(2u)	
(Jakobsson *et al*. 2010)	DNA Inhibitor and Protein Synthesis Inhibitor	↓	D	↓(p, 2g)	—	↑(1g)↓(1g)	—	↑	—	∼D	—	—	—	—	↑
(Dethlefsen and Relman 2011)	DNA Synthesis Inhibitor	↓*	D	—	↑(≥4u)↓(≥13u)	↑(≥7u)↓(≥8u)	↓(≥2u)	↑	—	D*	—	—	—	—	—
(Morotomi *et al*. 2011)	Cell Wall Synthesis Inhibitor or Protein Synthesis Inhibitor		D	—	↑(1g)	↑(2g)	—			—	—	—	—	—	
(Bajaj *et al*. 2013)	RNA Synthesis Inhibitor		∼D*	—	—	↑*(1f)↓*(1f)	—								
(O'Sullivan *et al*. 2013)	Various	∼↓	—	|*(1g)↓*(1g)	↑*(p)	|*(1f, 5g)↑*(8g)↓*(p, 1f, 5g)	|*(1g)↑*(1f, 1g)↓*(p)								
(Pérez-Cobas *et al*. 2013b)	Cell Wall Synthesis Inhibitor	↓	D	—	↑(6u)	↑(1u)↓(4u)	—	∼↑	—	—	↓(2g)	↓(1g)	↓(4g)	↓(1g)	—
(Arat *et al*., 2015)	Targets Bacterial Peptide Deformylase	↓	D	↑*(3u)	↓*(5u)	↑*(1u)↓*(7u)	↑*(2u)	↑*							
(Ladirat *et al*. 2014)	Cell Wall Synthesis Inhibitor			↓*(1g)	↑*(1u)	↓*(14-22u)	↑*(5u)				—	|(1g)	—	—	
(Panda *et al*., 2014)	Various	↓*	D	—	↑*(p, 1c, 1o, 1g, ≥12u)	↑*(2u)↓*(p, 1u)	—								
(Abeles *et al*. 2015)	Various	↓*	∼D	—	∼↓(p)	∼↑(p)	—	↑							
(Heinsen *et al*. 2015)	Protein Synthesis Inhibitor	↓*	D*	—	—	↑*(1u)↓*(22u)	↑*(1u)		—	D*	—	—	↓*(3u)	—	
(Rashid *et al*. 2015)	DNA Synthesis Inhibitor or Protein Synthesis Inhibitor	↓*		↓(1t)	↑*(1g)	↓*(1g)↓(2t)	↓(1s)		—^i^		—^i^	—^i^	—^i^	—^i^	
(Stewardson *et al*., 2015)	Various	∼↓	D*	↓*(p, 1c, 1o, 1f, 1g)	↑*(p, 1c, 1o, 1f, 1g, 1u)↓*(2f, 2g, 2u)	↑*(1f, 4g, 5u)↓*(2f, 2g, 10u)↕*(1g, 1u)	—		—	D*	↕*(p, 1c)	↓*(2f, 2g, 5u)	↑*(1c, 1f, 1g, 5u)↓*(2g, 2u)	—	
(Willmann *et al*. 2015)	DNA Synthesis Inhibitor	∼↓	D	—	—	—	↓(p)	D	—	∼D	—	—	—	—	∼D
(Zaura *et al*. 2015)	Various	↓*	D*	—	↑*(1g)	↓*(3g)	↓*(1g)	—	↓*	—	—	—	—	—	
(Raymond *et al*. 2016)	Cell Wall Synthesis Inhibitor	∼↓		↓*(1c, 2f, 3g, 8s)	↑*(1g, 1s)↓*(7s)	↑*(2g, 8s)↓*(1c, 2f, 11g, 23s, 2ss)	↓*(2f, 3g, 2s)	↑*	—		↓*(1f, 1g)	—	↓*(1f, 3s)	↓*(1f)	—
(Wipperman *et al*. 2017)	Combination	↓*	D*	↓(1g)	↑(1g)	↑(1g)↓(4g)	—		↑*	∼D*	—	↑(1g)↓(1g)	↑(3g, 1s)	↑(1g, 1s)	
(MacPherson *et al*. 2018)	Cell Wall Synthesis Inhibitor	↓*	D	↑*(1u)↓*(3u)	↑*(5u)	↑*(24u)↓*(95u)	↑*(1f, 3u)	↑*	—	—	—	—	**↑(3u)**	—	—
(Suez *et al*. 2018)	DNA Synthesis Inhibitor and DNA Inhibitor	↓*	D*	↑*(3g)↓*(1g)	↓*(5g, 6s)	↑*(6g, 4s)↓*(9g, 8s)	↑*(1g)↓*(1g)		— ^i^	D*					
(Dubinsky *et al*. 2020)	DNA Synthesis Inhibitor and/or DNA Inhibitor	↓*	D*	↑*(3u)↓*(10u)	↑*(1u)↓*(12u)	↑*(10u)↓*(45u)	↑*(6u)↓*(4u)	↑*							

Any effect shown within any subset of data within a study is presented here; for a more detailed summary, see Supplementary Table 1. ‘↑’ Indicates a positive effect on a variable, ‘↓’ indicates a negative effect on a variable, ‘|’ indicates a demonstrated effect of unclear direction, ‘↕’ indicates both a positive and negative effect shown according to different treatments or measurements, and ‘∼’ indicates a ‘possible’ or ‘slight’ effect. ‘D’ indicates clear disturbance of composition from baseline conditions or a control group. A blank space indicates that a variable was not measured by that study, and '—' indicates that the variable was measured, but no effect was found. Any entry in **bold**, with an asterisk ‘*’ was judged as significant by the authors of the study in question.

a ‘Treatment Mechanism’ column shows the mode of action of the antibiotic used in the study.

b ‘Short Term Effects’ columns show effects during, or immediately following, antibiotic treatment.

c ‘Long Term Effects’ columns show effects ranging between 14–1460 days after treatment ends.

d ‘Div’ columns show the effects on the diversity of the samples, according to metrics such as richness, Shannon Index, and Simpson Index

e ‘Com’ columns show the effects on the community composition of the samples, summarised by methods such as Bray-Curtis Dissimilarity, UniFrac Distances, and Principal Component Analysis.

f ‘Taxa’ columns show the effects on specific taxa in the in the samples, split into columns for each major phyla (ACT: Actinobacteria, BAC: Bacteroidetes, FIR: Firmicutes, and PRO: Proteobacteria), and listed following the codes: ‘t’ unclear taxa, ‘p’ phylum, ‘c’ class, ‘o’ order, ‘f’ family, ‘g’ genus, ‘s’ species, ‘ss’ subspecies and ‘u’ Operational Taxonomic Unit (OTU). The identities of taxa were confirmed using the List of Prokaryotic Names with Standing in Nomenclature (Parte 2018).

g ‘ABR’ columns show the effects on antibiotic resistance phenotypes or genes in samples.

h Result specifically for *Bacteroides* species.

i Results showing no clear effect were found at the end of study, but meaningful effects were measured at previous time points that would also be considered ‘long term’ as detailed in Table S1 (Supporting Information).

**Table 2.** Details of studies that address the effects of antibiotic treatment on the human gut microbiota.

Reference	Country	N Subjects^a^	N Samples^b^	Treatment^c^	Length^d^
(De La Cochetière *et al*. 2005)	France^e^	6 [0]	1; 1–4; 2	AMX, 3×500 mg [5 days]	55
(Jernberg *et al*. 2007)	Sweden^e^	4 [4]	1; 1; 7	CLI, 4×150 mg [7 days]	∼730
(Dethlefsen *et al*. 2008)	USA^e^	3 [0]	2-4; 1–2; 2	CIP, 2×500 mg [5 days]	175
(Jakobsson *et al*. 2010)	Sweden^e^	3 [3]	1; 1; 2	MTR, 2×400 mg; CIP, 2×250 mg; OME, 2×20 mg [7 days]	∼1460
(Dethlefsen and Relman 2011)	USA	3 [0]	11; 5; 20–24; 4–5; 10–12^f^	CIP, 2×500 mg [5 days]	37-103
(Morotomi *et al*. 2011)	Japan	5 [29]	0-1; 0–1; 0–2	Various, 150–3000 mg [1-8 days]	0-20
(Bajaj *et al*. 2013)	USA	20 [0]	1; 1; 0	RFX, 2×550 mg [∼56 days]	0
(O'Sullivan *et al*. 2013)	Ireland	42 [143]^g^	1^g^	Various, Not Specified	≤31
(Pérez-Cobas *et al*. 2013b)	Germany	1 [0]	1; 4; 1	SAM + CFZ, Not Specified [14 days]	26
(Arat *et al*. 2015)	USA^e^	46 [15]	1; 1; 0	GSK, 500–3000 mg [1-4 days]	0
(Ladirat *et al*. 2014)	Netherlands	10 [0]	2; 2; 0–4	AMX, 3×375 mg [5 days]	21
(Panda *et al*. 2014)	Spain^e^	21 [0]	1; 1; 0	Various, Not Specified [7 days]	0
(Abeles *et al*. 2015)	USA^e^	4 [5]	0; 3; 0	Various, Not Specified [≥42 days]	0
(Heinsen *et al*. 2015)	Germany^e^	5 [0]	1; 1; 1	PMM, 4000 mg [3 days]	43
(Rashid *et al*. 2015)	Sweden^e^	19 [10]	1; 1; 3–4	CIP, 2×500 mg; CLI 4×150 mg [10 days]	∼356
(Stewardson *et al*. 2015)	Switzerland	22 [20]	1; 1; 1	Various, Various [Not Specified]	∼28
(Willmann *et al*. 2015)	Germany	2 [0]	1; 3; 2	CIP, 2×500 mg [6 days]	28
(Zaura *et al*. 2015)	UK and Sweden	43 [23]	1; 1; 4	Various, Various [5-10 days]]	∼356
(Raymond *et al*. 2016)	Canada	18 [6]	1; 1; 1	CPR, 2×500 mg [7 days]	90
(Wipperman *et al*. 2017)	Haiti	38 [101]^g^	1^g^	HRZE, Not Specified [≥∼183 days]	34-1202
(MacPherson *et al*. 2018)	Canada^e^	70 [0]	1; 1; 2	AMX, 875 mg; CLA 125 mg [7 days]	∼14
(Suez *et al*. 2018)	Israel^e^	21 [25]	7; 7; 14	CIP, 2×500 mg; MTR, 3×500 mg [7 days]	180
(Dubinsky *et al*. 2020)	Israel	33 [16]	75 / 159^h^	Various, Various, [14-4646 days]	Various

a ‘N subjects’ column shows the number of treated subjects covered by the study, followed by the number of untreated controls in square brackets.

b ‘N samples’ column shows the number of samples taken from each subject, with samples taken before, during and after treatment separated by semicolons.

c ‘Treatment’ column shows the antibiotic treatment administered to the subjects, listing first the drug, then the daily dose, then the length of the course in square brackets. Drug codes used are: AMX/Amoxicillin, CLI/Clindamycin, CIP/Ciprofloxacin, MTR/Metronidazole, OME/Omeprazole, RFX/Rifaximin, SAM/Ampicillin-Sulbactam, CFZ/Cefazolin, GSK/GSK1322322, PMM/Paromomycin, CPR/Cefprozil, HRZE/Isoniazid-Rifampin-Pyrazinamide-Ethambutol, CLA/Clavulanic Acid.

d ‘Length’ column shows the length of the study, shown as the number of days between the end of treatment and the last sample collected.

e Location inferred from author locations and locations of ethical approval.

f Study encompassed two courses of antibiotic treatment, and samples are listed in the format: before treatment; during first treatment; interval; during second treatment; after treatment.

g Study used cross-sectional experimental design, so each subject was only sampled once.

h Study collected a widely different number of samples from each subject, for a total of 75 antibiotic associated and 159 non-associated samples.

## ECOLOGICAL AND EVOLUTIONARY EFFECTS

Accurately measuring changes in the ecology and evolution of any community is a major challenge, let alone a microscopic community which is largely inaccessible without medical intervention such as the gut microbiota. Antibiotic treatment may well affect every aspect of the community, including the flow of nutrients and energy, the rates and mechanisms of gene transfer, and the strength of selection for different traits and lifestyles, but the simplest effect is likely to be the direct suppression of certain bacterial populations. Therefore, the most basic measurement to compare between antibiotic treated and untreated communities is the abundance of different bacterial populations in the microbiota.

### Absolute and relative abundance

An important distinction to make when discussing the abundance of bacterial populations is between absolute abundance—the actual number of cells belonging to a taxon in a given sample—and relative abundance—the *proportion* of a sample that is comprised of any given taxon (Shanahan and Hill 2019). In several respects, absolute abundance is the more meaningful metric. The actual density of cells has the potential to affect many important aspects of microbial ecology including competition for resources and bacteria-phage interactions, and is a fundamental component of quorum sensing systems, which can influence biofilm formation, virulence and sporulation (Bassler and Losick 2006). Absolute abundance is also relevant to understanding evolutionary processes within the gut microbiota as mutation supply rate is proportional to population size and this will impact a microbial population's adaptive response to natural selection (Hall *et al*. 2010). Moreover, smaller populations are more susceptible to genetic drift, which could potentially arise from bottlenecking events following antibiotic treatment. Aside from missing these details, a problem with relative abundance measurements is that they introduce ambiguity into changing population sizes, since it is impossible to know whether any given species has truly, for example, increased in abundance, or if it has merely maintained a stable population while other taxa have diminished (Harrison *et al*. 2021). Unfortunately, the most common methods used to investigate the microbiota in the reviewed studies, 16S rRNA sequencing and shotgun metagenomic sequencing, only provide relative abundance data. Despite this, several studies measured the effects of antibiotic treatment on the absolute abundance of the gut microbiota through other methods. The most common was quantitative PCR, which led to inconsistent results between studies. Two studies found clearly lower total microbial abundances in the microbiome during antibiotic treatment compared to samples taken before or after treatment (Suez *et al*. 2018, Dubinsky *et al*. 2020). In contrast, Ladirat *et al*. found no significant effect of treatment on the total microbial abundance (Ladirat *et al*. 2014), while Panda *et al*. actually found that the total microbial abundance increased after treatment, particularly for beta-lactam treated patients (Panda *et al*. 2014), which perhaps reflected a major overgrowth of resistant species. Other studies directly cultured portions of their samples to measure the absolute abundance of certain groups, and found decreased abundance during treatment in some taxa but not universally (O'Sullivan *et al*. 2013; Rashid *et al*. 2015). The importance of measuring absolute abundances is increasingly recognised in microbiome science, and there have been several recent calls for wider adoptions of a range of methods, including flow cytometry (Vandeputte *et al*. 2017), spiking samples with strains of known abundance for comparison (Harrison *et al*. 2021), or even comparing bacterial gene proportions to the relatively stable proportions of human and viral genes in samples (Dubinsky *et al*. 2020). Through these methods and more, the measurement of absolute abundances will allow for far more accurate and thorough ecological investigation of the antibiotic-treated gut microbiota in future studies.

Although relative abundance provides less information than absolute abundance, it still has considerable merit. The proportions of different taxonomic groups in a community, otherwise known as the community composition, can be compared over time to determine which groups are affected in which ways by antibiotic treatment. The vast majority of identified bacterial species in the gut microbiota belong to just a few phyla, namely the Actinobacteria, Bacteroidetes, Firmicutes and Proteobacteria (Rajilić-Stojanović and de Vos 2014; Rinninella *et al*. 2019). Phyla are very high-level divisions of organisms, each encompassing enormous diversity, but it is still possible to observe some broadly common patterns in the reviewed studies. Most clearly, many of the detected effects on Actinobacteria relative abundance are negative, although some more recent studies have found a combination of positive and negative effects, perhaps owing to more sensitive modern techniques (Table 1). The negative effects were often attributed to organisms in the genus *Bifidobacterium* (Table S1, Supporting Information), a well-known group of bacteria which metabolise a wide range of carbohydrates and are used in commercial probiotics for their reputed beneficial health effects (Pokusaeva, Fitzgerald and van Sinderen 2011). Conversely, for the Bacteroidetes phylum, also characterised by extensive carbohydrate metabolism (Thomas *et al*. 2011), many detected effects on relative abundance were positive, although this trend was less clear than the reduction of Actinobacteria (Table 1). These effects were often reported for the *Bacteroides* genus, but other genera such as *Alistipes, Parabacteroides* and *Prevotella* were also affected in some studies (Table S1, Supporting Information). Effects relating to the relative abundance of organisms in the Firmicutes phylum were reported more often than for any other group (Table 1), which is not surprising for the most abundant and diverse group of bacteria in the gut (Rajilić-Stojanović and de Vos 2014). Many positive and negative effects were found, but in most cases more organisms were suppressed than amplified. Within this phylum, the *Faecalibacterium* genus, a major producer of the colonic energy source butyrate (Ferreira-Halder *et al*. 2017), was often described as reduced, as was the *Blautia* genus, while *Enterococcus* was amplified in several instances (Table S1, Supporting Information). Effects on the relative abundance of Proteobacteria showed no prevailing direction but were detected less commonly overall (Table 1), reflecting the group's lower overall abundance in the gut. Rare effects on other taxa have also been demonstrated (Table S1, Supporting Information), and include, for example, increases in the relative abundance of archaeal phylum Euryarchaeota (O'Sullivan *et al*. 2013). Proteobacteria and Bacteroidetes are gram-negative bacteria, while Actinobacteria and most Firmicutes are gram-positive (Rajilić-Stojanović and de Vos 2014), which may go some way to explain their responses to antibiotics, particularly those that affect the cell wall (Table 1). However, it is not always clear why different groups of species are amplified or suppressed by antibiotic treatment.

It is intuitive that many of the suppressed groups are directly inhibited by the antibiotics, and some information exists on the sensitivity of certain gut species to certain antibiotics (van Schaik 2015), but there are also huge gaps in our understanding of this area. Some of the amplified species are likely to be resistant organisms filling the empty niches of inhibited species, or even exploiting freed resources from the dead cells of targeted bacteria, but as these changes progress through the network of ecological interactions, they are likely to lead to the amplification and suppression of species not directly affected by antibiotics. Complicating matters further, large differences in the composition of the microbiota of individuals sampled at different time points during treatment have been found (Pérez-Cobas *et al*. 2013a); this finding suggests that antibiotic treatment may induce a series of cascading changes in the gut microbiota, rather than simply flipping it from an undisrupted state to a disrupted state. In a few anecdotal cases, clear mechanisms behind important demographic shifts have also been shown such as the widespread amplification in one study of *Lachnoclostridium boltae* relative abundance that coincided with the enrichment of antibiotic resistance genes in this species (Raymond *et al*. 2016). Similarly, another study found that members of the Enterobacterales and Lactobacillales orders with at least one fluoroquinolone resistance allele were highly dominant in the microbiota of antibiotic treated subjects, and far less relatively abundant in untreated subjects (Dubinsky *et al*. 2020). However, cases where we understand the mechanistic basis for antibiotic mediated effects on the ecology of the gut microbiome are still the exception rather than the rule.

### Effects on community composition

**Figure 1.**
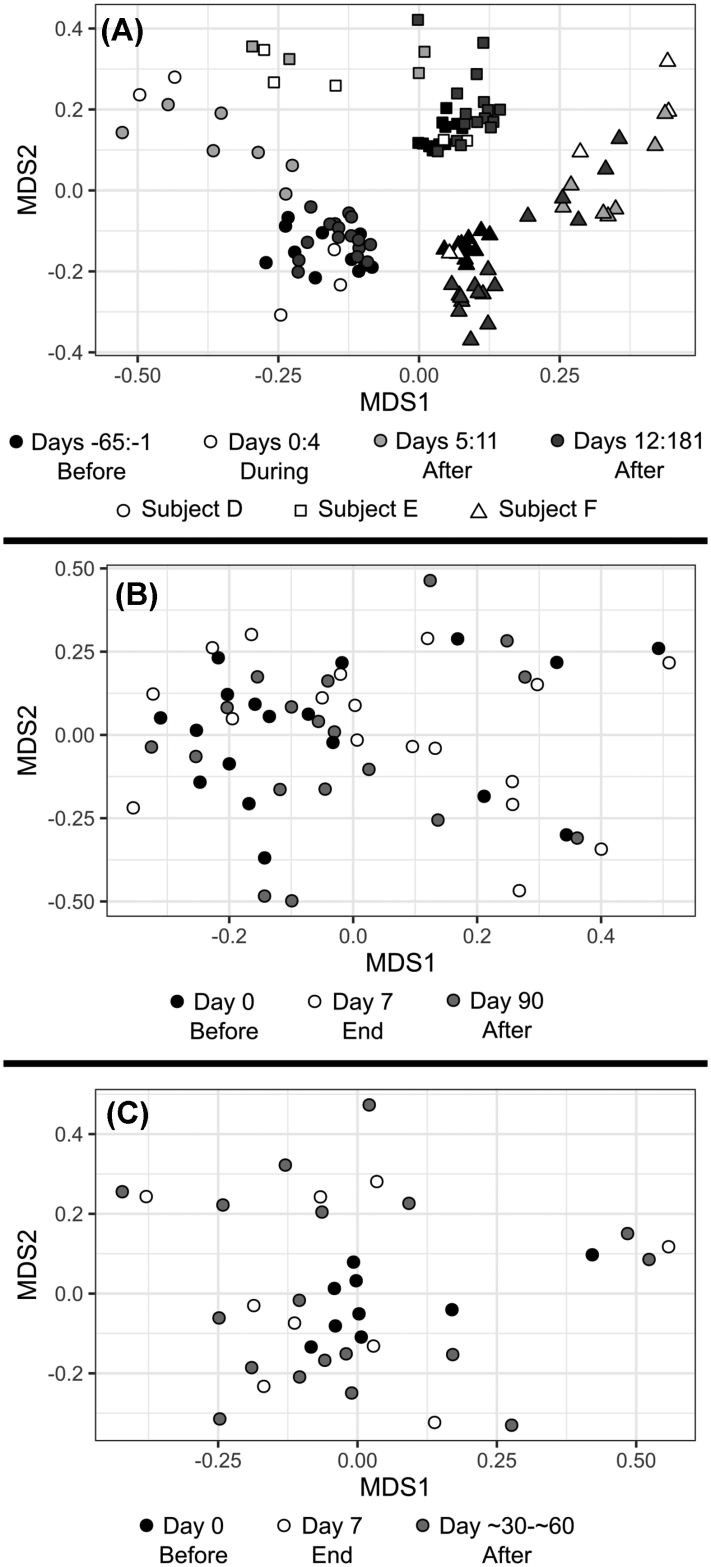
Example effects of antibiotic treatment on the community composition of the gut microbiota. All graphs show the relationship between samples via non-metric multidimensional scaling of Bray-Curtis dissimilarity. Data for all graphs was processed and visualised using the 'vegan' package and the 'ggplot2' package in the R programming language (Wickham 2016; Oksanen *et al*.^2019^; R Core Team 2020). **(A)** shows data from only the first round of treatment covered by Supplementary Dataset 1 of Dethlefsen & Relman., representing up to 40 samples from each of 3 subjects treated with ciprofloxacin, with a stress of 0.156849 (Dethlefsen and Relman 2011). **(B)** shows data from only the treated subjects covered by Raymond *et al*. and taxonomically classified by Supplementary Data 1 of Chng *et al*., representing 18 subjects treated with cefprozil, and with a stress of 0.2342019 (Raymond *et al*. 2016; Chng et al. 2020). **(C)** shows data from only the clindamycin treatments covered by Zaura *et al*. and taxonomically classified by Supplementary Data 1 of Chng *et al*., representing 9 subjects, and with a stress of 0.1898547 (Zaura *et al*. 2015; Chng *et al*. 2020).

Below the level of phyla, the collected information on each family, genus, or species in the gut microbiota becomes increasingly vast, making it difficult to determine the true scale of disturbance, or draw meaningful conclusions between studies. Furthermore, it has been shown that the gut microbiota can fluctuate naturally over time (Dethlefsen and Relman 2011), so the effects of antibiotic treatment must be separated from normal variation. Here, various methods exist to condense complex multidimensional data, such as community composition into, for example, distance and dissimilarity metrics, allowing easy comparison of different communities over time or according to different treatments (Bray and Curtis 1957; Lozupone and Knight 2005; Jolliffe and Cadima 2016). These comparisons can also be made in cases where studies didn't directly measure the abundance of any taxa, by substituting molecular fingerprints for the full community composition (De La Cochetière *et al*. 2005; Jernberg *et al*. 2007). Some methods even incorporate phylogenetic distance into the equation (Lozupone and Knight 2005), or highlight which groups of species most underlie the divergence between communities (Jolliffe and Cadima 2016). Through these methods, the vast majority of reviewed studies determined that their subjects’ microbiota were truly disrupted directly following antibiotic treatment (Table 1), and studies that took several samples before treatment to establish a baseline level of variation (Dethlefsen *et al*. 2008; Dethlefsen and Relman 2011; Ladirat *et al*. 2014; Suez *et al*. 2018), or compared their treated subjects to untreated controls (Jernberg *et al*. 2007; Jakobsson *et al*. 2010; Morotomi *et al*. 2011; Raymond *et al*. 2016; Suez *et al*. 2018), showed that this disturbance was distinct from normal fluctuations. After antibiotic treatment, these same metrics showed that composition tended to return towards the pre-treatment state, and fewer studies detected a meaningful disturbance of the community by the end of their sampling period (Table 1), demonstrating the resilience of the gut microbiota community. For example, Fig. 1A shows the microbiota composition of each of the three subjects in Dethlefsen and Relman's study diverging from baseline during and immediately after treatment, then progressing back towards their initial states (Dethlefsen and Relman 2011). Such recoveries could be caused by consistent selection pressures imposed by the nutritional and host environment favouring the re-assembly of a similar community, more complex negative-frequency dependent dynamics where relationships between species encourage less common members to return to higher abundance (Lozupone *et al*. 2012), or the existence of spatial refuges in the gut that are less strongly affected than faecal samples reflect, from which depleted species can recolonise after treatment. One study investigated these recoveries to baseline composition in detail and found that recovery was faster and more complete when supplemented by a personal faecal microbiome transplant, but actually less complete when supplemented with a probiotic treatment (Suez *et al*. 2018). Regardless, this tendency towards resilience comes with some important caveats.

In several studies where the community composition generally returned to baseline, some individual subjects completely failed to recover (De La Cochetière *et al*. 2005; Dethlefsen and Relman 2011; Raymond *et al*. 2016; MacPherson *et al*. 2018). For example, in Dethlefsen and Relman's study, 2 out of 3 subjects mostly recovered their initial composition after a week, while the third took several months to settle into a stable state which was still noticeably different from the outset (Dethlefsen and Relman 2011). In Raymond *et al*.’s study, taxonomic composition alone showed no clear response to antibiotic treatment (Fig. 1B) (Raymond *et al*. 2016). However, a more complex analysis that included other factors such as resistance genes and sequencing depth showed that even though 22 out of 24 subjects broadly returned to their initial states after several months, the microbiota of 2 other subjects remained dramatically altered. One subject had a dramatically reduced relative abundance of the *Prevotella* genus which had previously dominated their microbiota whilst another saw large blooms in the relative abundance of Enterobacteriaceae and Verrucomicrobiaceae families. These examples could be related to other lifestyle changes—in fact one of the affected subjects in the latter study was receiving concurrent ferrous sulphate treatment, which can have effects on the microbiota in rats (Dostal *et al*. 2012)—but antibiotic treatment cannot be ruled out as the cause. Furthermore, even in subjects where the gut microbiota broadly recovered, persistent effects on some taxa sometimes remained (Table 1). For example, Jernberg *et al*. found that while the overall composition of their subjects’ microbiota had mostly recovered a month after treatment, the composition of strains in the *Bacteroides* genus did not recover, even up to 2 years post treatment (Jernberg *et al*. 2007). Similarly, Dethlefsen *et al*. showed that a Clostridiales species that was present in all samples from two subjects before treatment was never observed again up to 175 days after treatment (Dethlefsen *et al*. 2008). It is also worth noting which particular studies found persistent disturbance in the gut community. Dethlefsen and Relman took far more samples from each of their subjects than any other study (Dethlefsen and Relman 2011), and Stewardson *et al*. used household contacts as paired controls for each amoxicillin-treated subject, along with one of the largest subject and control pools of the longitudinal studies (Stewardson *et al*. 2015) (Table 2). Both of these studies found statistically significant, long-term disturbance in the composition of their subject's gut microbiota, suggesting that cryptic disturbance may have remained undetected in many of the other studies with smaller sample sizes (Tables 1 and 2).

### Effects on diversity

**Figure 2.**
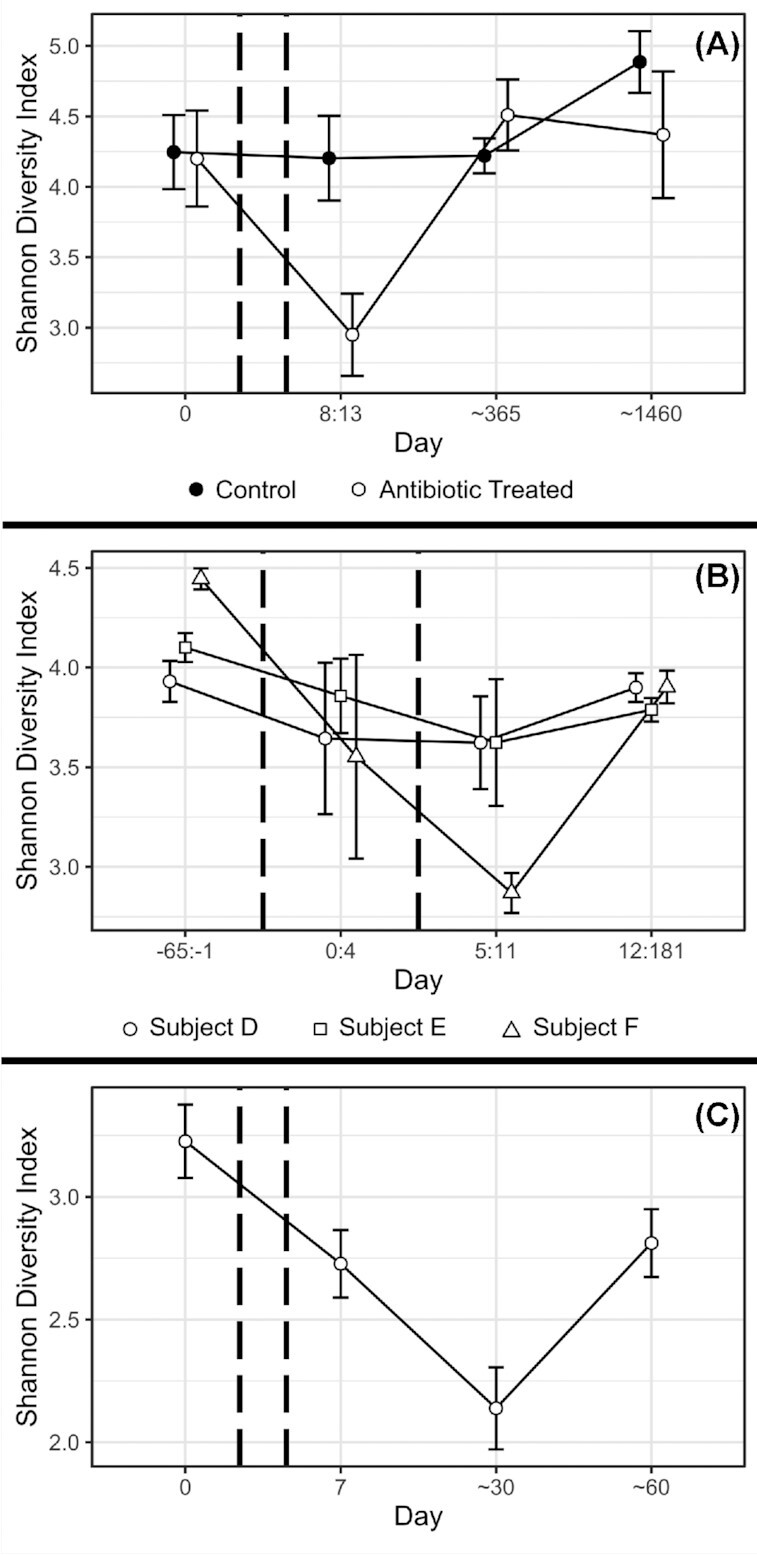
Example effects of antibiotic treatment on diversity of the gut microbiota. In all graphs, antibiotic treatment took place between pairs of dashed vertical lines, and error bars represent 1 standard error around the mean. Data for all graphs was processed and visualised using the 'vegan' package and the 'ggplot2' package in the R programming language (Wickham 2016; Oksanen *et al*.^2019^; R Core Team 2020). **(A)** Shows data from Table 5 (Supporting Information) of Jakobsson *et al*., representing three untreated controls and three subjects treated with a combination of two antibiotics (metronidazole and clarithromycin) and a proton pump inhibitor (omeprazole) (Jakobsson *et al*. 2010). **(B)** Shows data from only the first round of treatment covered by Supplementary Data set 1 of Dethlefsen & Relman, representing up to 40 samples from each of 3 subjects treated with ciprofloxacin (Dethlefsen and Relman 2011). **(C)** Shows data from only the clindamycin treatments covered by Zaura *et al*. and taxonomically classified by Supplementary Data 1 of Chng *et al*., representing nine subjects (Zaura *et al*. 2015; Chng *et al*. 2020).

Diversity is a commonly used metric that has been defined in several ways (Jost 2006), but usually incorporates how many species are present in a community—the species richness—and how balanced their populations are—species evenness. In the reviewed studies, diversity was described by several metrics (Table S1) including the Shannon Index (Spellerberg and Fedor 2003; Jost 2006), the Gini-Simpson Index (Jost 2006), estimated total richness (Chao 1984; Chao 1987), or simply the detected species richness, which all measure slightly different phenomena, but are useful for comparisons within individual studies. One of the most consistent results across all reviewed studies was that the gut microbiota's diversity dropped in response to antibiotic treatment (Table 1). Furthermore, even more reliably than the recovery of composition, the diversity of the gut microbiota was found to return to normal levels following antibiotic treatment (Table 1), and was even significantly increased in one study for previously-treated subjects compared to controls (Wipperman *et al*. 2017). Fig. 2 shows representative examples broadly reflecting this pattern. However, once again, this apparent recovery might not tell the whole story as 16S rRNA amplicon sequencing, used by many of the reviewed studies, is largely unable to reliably identify organisms at taxonomic resolutions below the genus level. In contrast, one study that paid particular attention to individual strains in the *Bacteroides* genus found that their diversity dropped dramatically following antibiotic treatment, and remained lowered even 2 years later (Jernberg *et al*. 2007). If this strain-level loss of diversity is the norm, the effects of antibiotic treatment on the diversity of the gut microbiota may be far more persistent than currently recognised. As methods for analysing molecular data become more advanced and more widely used (Truong *et al*. 2017), it should become clear whether long-term disturbance that has been observed at the strain level (Jernberg *et al*. 2007) represents a broad pattern.

Another ecological factor to consider is the heterogeneity between gut microbiota samples, or in other words how dissimilar samples taken from different subjects are to one another. This is sometimes called beta diversity (in contrast to alpha diversity which describes diversity *within* individual samples), but beta diversity has been inconsistently defined since its conception (Whittaker 1960) and recent work argues the term should be reserved for describing a specific relationship between the number of species found in individual samples and the total sample pool (Tuomisto 2010), so the more general term heterogeneity is more useful here. The effect of antibiotic treatment on the heterogeneity of gut microbiota samples wasn't a major focus of any of the reviewed studies, but through their measurements of dissimilarity and distance between samples (Bray and Curtis 1957; Lozupone and Knight 2005; Jolliffe and Cadima 2016) it is possible to make some observations. In several cases, antibiotic treated gut microbiota seemed to be less similar to each other than untreated gut microbiota (Abeles *et al*. 2015; Zaura *et al*. 2015; Raymond *et al*. 2016; MacPherson *et al*. 2018) (Fig. 1C), representing an increase in heterogeneity between individuals during treatment. However, the opposite pattern was show in Arat *et al.’s* study, where treated communities clustered together more tightly than untreated ones (Arat *et al*. 2015). Heterogeneity or dissimilarity between subjects is more difficult to meaningfully interpret than diversity within subjects, but may still offer important insights into the effect of antibiotic treatment. While there are many differences between the composition of the gut microbiota between humans, gut microbiota samples in general seem to be less heterogeneous than microbiota samples from other body locations (Huttenhower *et al*. 2012), and this likely reflects the broadly similar niche offered by the human gut microbiome regardless of the individual. If a uniformly administered antibiotic treatment leads to a decrease in the similarity between different subjects' microbiota compositions, it suggests that this niche is being disrupted, and moreover that the treatment is affecting different subjects in different ways, perhaps introducing stochastic effects, or exacerbating existing differences between subjects. It is also important to remember that a single subject's microbiota is not an ecologically isolated unit, but rather a community that is subject to migration between other microbiomes and the wider environment (Song *et al*. 2013; Martínez *et al*. 2015; Asnicar *et al*. 2017). Ecological measurements which consider not just individual communities but the meta-community as a distinct structure, are important reflections of this fact. It would be valuable for studies to investigate this factor more directly in the future, perhaps using multiple cohorts of related subjects to test whether antibiotic treatment leads to predictable changes in within-cohort heterogeneity, and maybe also using true beta diversity (Tuomisto 2010) as a measurement of this.

### Other ecological effects

A few reviewed studies investigated further aspects of the gut microbiota's ecology in response to antibiotic treatment. Heinsen *et al*. compared the effects of antibiotic treatment in the lumen and the mucosa of the colon, which are known to support different bacteria (Mark Welch *et al*. 2017; Flynn *et al*. 2018), and found that while taxa differed between the two environments, their responses to antibiotic treatment were broadly similar (Heinsen *et al*. 2015). Several studies also measured some indication of the metabolic activity of the gut microbiota and highlighted some interesting trends including a sharp increase in certain kinds of phospholipids towards the end of treatment (Pérez-Cobas *et al*. 2013b) and significant increases in a variety of fatty acids in the blood serum following treatment (Bajaj *et al*. 2013). Of particular note, increased levels of faecal succinate, monosacharrides, and oligosaccharides were found during treatment in one particular study which led the authors to speculate that these metabolites, normally used as energy sources by members of the gut microbiota, were less efficiently utilised and that metabolic activity was disrupted by antibiotic treatment (Ladirat *et al*. 2014). The gut microbiota's resistance to invasion may also be affected by antibiotic treatment. It has long been suspected that the gut microbiota is more vulnerable to invasion by external microbes after antibiotic treatment (Ferrer *et al*. 2017). This theory may be supported by Suez *et al*.’s results showing that a probiotic cocktail was far more successful at colonising antibiotic-treated subjects than untreated controls (Suez *et al*. 2018).

Another major area that deserves more focus is the ecology of non-bacterial members of the gut microbiota. Some archaea, such as *Methanobrevibacter smithii* (Dridi *et al*. 2009), and various eukaryotes, such as yeasts and *Blastocystis spp*. (Scanlan and Marchesi 2008; Scanlan *et al*. 2014), are common residents of the human gut. But perhaps some of the most abundant and diverse inhabitants are the viruses, particularly bacteriophages, which infect bacterial populations and can engage in dynamics similar to predator-prey cycles with their hosts (Sutton and Hill 2019). Only one of the reviewed studies played particular attention to viral ecology and although they did not observe a very clear effect of antibiotic administration on the diversity or overall composition of viruses they found that viral homologues putatively assigned to Firmicutes hosts were significantly increased after treatment and those related to Bacteroidetes hosts decreased, matching the shifts in bacterial populations in that study (Abeles *et al*. 2015). The ecology of bacteriophages is inherently linked to the ecology of bacteria, so even if antibiotic treatment doesn't affect bacteriophages directly, they are likely to be influenced in complex ways.

One particularly important factor in phage ecology is the difference in life-cycles between virulent and lysogenic phages. Virulent, or lytic, phages bind to and replicate within host cells, which they ultimately destroy upon the release of infectious virions into the environment. However, lysogenic phages can either complete a lytic cycle as described above or can be incorporated into the host's genetic material and reside there indefinitely, replicating along with the bacterium's natural life cycle (Sutton and Hill 2019). As lysogenic phages can alternate between these two strategies, and both have varying effects on the ecology and evolution of microbial populations, the factors that cause lysogens to be induced and complete a lytic cycle are of considerable interest (Knowles *et al*. 2017; Sutton and Hill 2019). It has long been known that antibiotics can trigger lysogen induction, and in fact mitomycin C is commonly used to test for the presence of lysogens in bacterial cultures (Otsuji *et al*. 1959), while other antibiotics such as fluoroquinolones have a similar effect (López *et al*. 2014). Based on these findings it is intuitive that antibiotic treatment is very likely to have an effect on the balance between lysis and lysogeny in intestinal phages with potential downstream effects on the ecological dynamics of bacterial populations in the gut microbiome.

### Separating ecology and evolution

Even if the composition of the community largely recovers after treatment, with some exceptions described above, this only means that the same groups of organisms are present before and after treatment. If evolutionary change has taken place, the fundamental nature of those organisms may have been altered during treatment, potentially leading to wide-reaching, functional changes that may not be immediately evident. To complicate matters, it is surprisingly difficult to pin down the distinction between ecological and evolutionary change, and even studies that explicitly focus on the interplay between ecology and evolution often lack precise definitions of the two (Pelletier, Garant and Hendry 2009; Schoener 2011; Hiltunen, Virta and Laine 2017; Kokko *et al*. 2017; Lowe, Kovach and Allendorf 2017). Most definitions of evolution involve changes in the proportions of alleles within specific populations, but bacterial populations in particular can be very hard to define, owing to their capacity for horizontal gene transfer and lack of sexual recombination (Konstantinidis, Ramette and Tiedje 2006; Cohan and Perry 2007; Rocha 2018). If evolution of different species in the gut is driven by natural selection, rather than selectively-neutral genetic drift, it is also important to explain how genetic changes affect the phenotype, and why that phenotypic change increases fitness. Investigating to this level of depth is highly labour intensive, so for the most part the evolution of the gut microbiota during antibiotic treatment is not as well understood as the ecology. However, in one particular, obviously relevant aspect—the evolution of antibiotic resistance—there has been some exploration.

### Effects on antibiotic resistance

**Figure 3.**
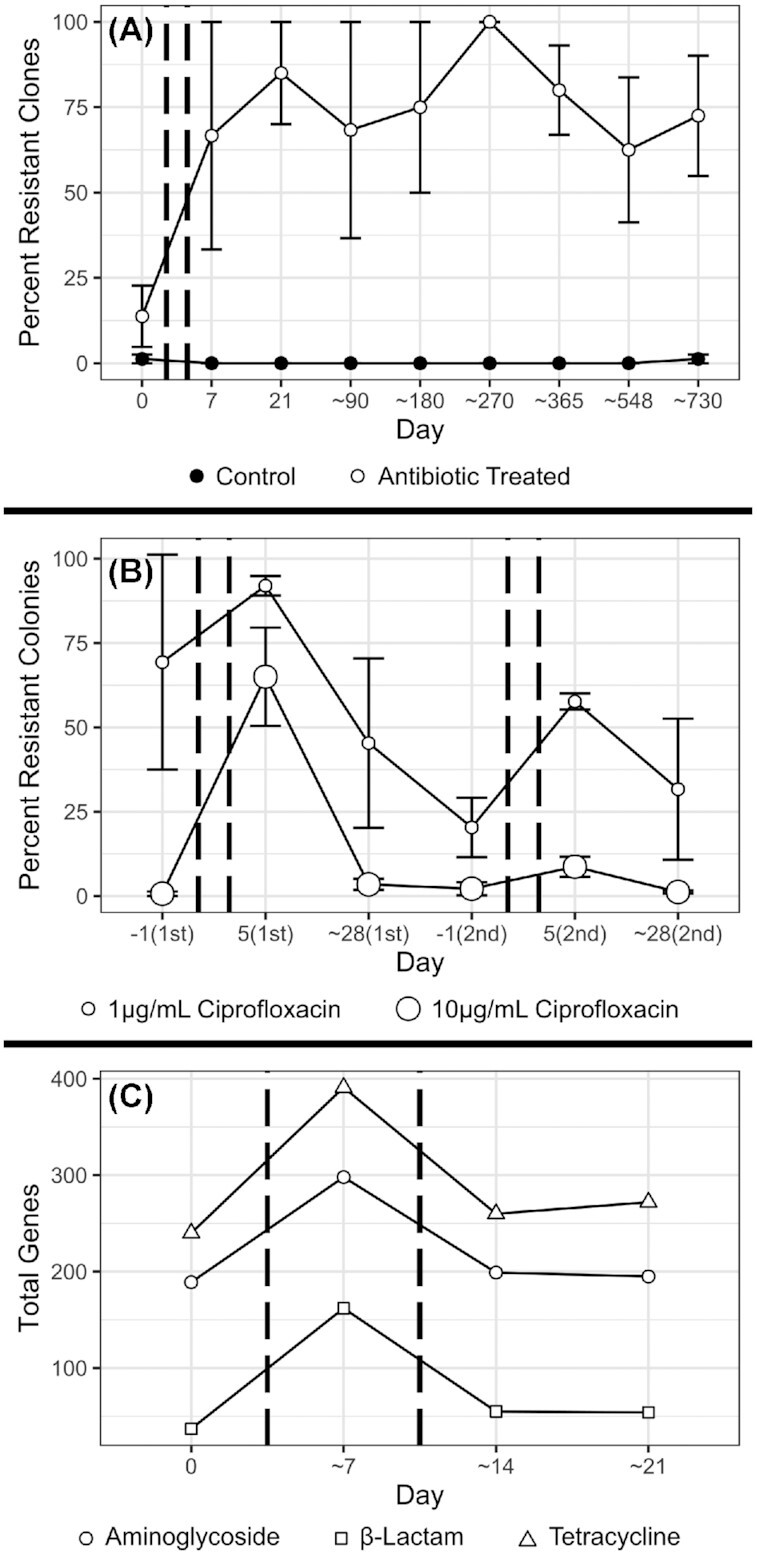
Example effects of antibiotic treatment on antibiotic resistance genes and phenotypes in the gut microbiota. In all graphs, antibiotic treatment took place between pairs of dashed vertical lines, and error bars, where present, represent one standard error around the mean. Data for all graphs was visualised using the 'ggplot2' package in the R programming language (Wickham 2016; R Core Team 2020). **(A)** shows data from Supplementary Figure 1 of Jernberg *et al*., representing the percentage of up to 20 *Bacteroides* clones from each of four untreated controls, and four subjects treated with clindamycin, which were not inhibited by 8 mg/L of clindamycin (Jernberg *et al*. 2007). **(B)** shows data from both rounds of treatment covered by Table 2 (Supporting Information) of Dethlefsen & Relman, representing the count of colonies grown on media supplemented with ciprofloxacin as a percentage of the count grown on media without ciprofloxacin, from 3 subjects treated with ciprofloxacin (Dethlefsen and Relman 2011). **(C)** shows data pooled from the placebo and probiotic treatments of Figure 7 of MacPherson *et al*., representing the total antibiotic resistance genes of 3 classes, detected in a microarray survey of samples from 70 subjects (MacPherson *et al*. 2018).

As expected, most reviewed studies showed a clear rise in antibiotic resistance genes or phenotypes following treatment (Table 1, Fig. 3), through various methods such as functional analysis of gene fragments, resistance gene microarrays, or culture work (Table S1). In some cases this rise was quite striking, such as in Jacobsson *et al*.’s study where a particular resistance gene (*erm*(B)) increased by up to 5 orders of magnitude above pre-treatment levels (Jakobsson *et al*. 2010). However, even this intuitive conclusion was not entirely simple, with other studies finding large inter-subject variation in their results (Pérez-Cobas *et al*. 2013a; Willmann *et al*. 2015) and in one case a clear decrease of a family of antibiotic resistance genes during treatment (Willmann *et al*. 2015). This could suggest that fitness trade-offs exist between different types of resistance, where resistance genes not corresponding to the type of antibiotic used in treatment are selected against in the competitive environment of the microbiome. On the other hand, there are several clear examples where types of antibiotic resistance were promoted beyond those directly corresponding to treatment (Pérez-Cobas *et al*. 2013a; Willmann *et al*. 2015; Raymond *et al*. 2016; MacPherson *et al*. 2018), such as increases in aminoglycoside and tetracycline resistance following beta-lactam treatment (MacPherson *et al*. 2018). In these cases, several resistance genes might be clustered together on the same genome and/or on the same mobile genetic element, and so resistance genes not corresponding to the treatment could gain prevalence by hitchhiking alongside directly beneficial genes. Alternatively, these genes might simply have wider effects than are currently understood. After treatment, some studies found that the levels of antibiotic resistance genes had returned to pre-treatment levels (*e.g*. Fig. 3B and C), but others found them to remain raised (*e.g*. Fig. 3A) as per the aforementioned elevated levels of the *erm*(B) gene that persisted for up to 4 years after treatment (Jakobsson *et al*. 2010).

These elevations in antibiotic resistance were mostly found at the community level, so they might not represent true evolution. Instead of populations evolving resistance in response to antibiotic induced selection, it might simply be the case that existing resistant species in the gut increase in abundance. Indeed, in keeping with previous research (van Schaik 2015), antibiotic resistance was regularly detected in reviewed studies even before treatment (Jernberg *et al*. 2007; Jakobsson *et al*. 2010; Willmann *et al*. 2015; Raymond *et al*. 2016; MacPherson *et al*. 2018). However, at least one study conclusively demonstrated the evolution of antibiotic resistance in their subjects’ gut microbiota (Jernberg *et al*. 2007). In this study, the levels of resistance in individual *Bacteroides* isolates were measured and isolates were discrimminated at the strain level using rep-PCR. Using this approach, it was found that not only did the total level of resistance increase, but crucially that in three of their 4 treated subjects, strains not previously shown to be resistant became highly resistant following antibiotic exposure (Jernberg *et al*. 2007). None of the other reviewed studies deeply investigated genetic change within individual strains. However, several recent studies have clearly demonstrated the occurrence of adaptive evolution in the microbiome in the absence of antibiotic administration (Garud *et al*. 2019; Zhao *et al*. 2019), which suggests that evolution by means of natural selection is very likely to occur in this same environment when the extreme selection pressure of antibiotic treatment is introduced. These studies also showcase some powerful methods of investigating evolution which could be applied to studies incorporating antibiotics. One study sequenced the full genomes of hundreds of isolates of a single species (*Bacteroides fragilis*) and used them to construct phylogenetic trees where they could track lineages and infer genetic changes relative to common ancestors (Zhao *et al*. 2019), and another used novel methods to narrow down large metagenomic data sets to a subset of cases where it was possible to reconstruct the genotype of a given species' dominant strain, and profiled genetic changes within these estimated dominant strains over time (Garud *et al*. 2019). These studies also employed a number of approaches for separating adaptive evolution from genetic drift, such as measuring the ratio between mutations which change the amino acid product of a nucleotide codon and those that cause no change, investigating whether the same mutations occur independently in multiple separate lineages, and comparing the rate of genetic change to models of neutral evolution (Garud *et al*. 2019; Zhao *et al*. 2019). To properly understand the evolutionary effects of antibiotic treatment on the microbiota, it will be crucially important for future studies to incorporate similar methods and measure genetic changes within populations of individual strains.

Intuitively, horizontal transfer of resistance genes has the capacity to confer a selective advantage during treatment, and could also explain rises in antibiotic resistance. Several studies have found evidence to support this hypothesis; for example, Abeles *et al*. found increases in the antibiotic resistance genes carried by transmissible viruses during treatment (Abeles *et al*. 2015), Raymond *et al*. found that the resistance gene *bla*_TEM-1_, which increased 90-fold during treatment in one subject, was located among genes known to belong to an *Escherichia coli* plasmid (Raymond *et al*. 2016), and Willmann *et al*. found that a promoted resistance gene resided close to markers of a *Bacteroides fragilis* mobile genetic element (Willmann *et al*. 2015). Horizontal gene transfer is possible between very distantly related bacteria (Amábile-Cuevas and Chicurel 1992), and therefore has the potential to spread resistance genes to new populations.

### Other evolutionary effects

Most evidence for evolution in the gut microbiota following antibiotic treatment focuses only on antibiotic resistance, but scattered evidence for other evolutionary change exists. Multiple studies show that treatment affects the presence of genes coding for efflux pumps in the gut microbiota (Pérez-Cobas *et al*. 2013a; Willmann *et al*. 2015), which can confer resistance to certain antibiotics by pumping the drugs out of the cell, but also interact with other elements of the microbiome environment such as the host immune system and bile (Piddock 2006). In the same way that many studies measured increases in the community-wide presence of antibiotic resistance genes in their samples, other groups of functional genes were found to change with treatment, although none so consistently as resistance. Several subjects in on particular study had increased levels of genes relating to sporulation and germination (Pérez-Cobas *et al*. 2013a), which allow certain species to produce resilient spores and survive through high-stress environments (Browne *et al*. 2016). Elsewhere, an increase in genes relating to fatty acid oxidation and vitamin biosynthesis, as well as a decrease in genes relating to conjugated bile acid biosynthesis have been noted (Wipperman *et al*. 2017). Evidence from outside of the gut shows that in response to fluoroquinolone exposure, *Enterococcus faecalis* may evolve a slower growth rate and *Pseudomonas aeruginosa* may produce increased amounts of quorum-sensing molecules, among other effects (Wassermann *et al*. 2016; Sun *et al*. 2018). Despite the dearth of direct investigation, evolution in a variety of traits seems very likely to occur in the gut microbiota during antibiotic treatment.

It is also well established, from examples outside of the gut, that antibiotics can affect several underlying mechanisms of the evolutionary process in bacteria. The presence of antibiotics can activate a stress response in some species that increases the rate of both horizontal-gene transfer and mutation (Andersson and Hughes 2014). Beyond this, recombination of the genome within and between cells can also be stimulated by antibiotics (López‐Camacho *et al*. 2007). All of these processes widen the pool of variation in a community that selection can then act upon. In these ways, antibiotic treatment may be able to influence the evolution of the gut microbiota not just by imposing selection pressures upon it, but by altering the mechanisms through which evolution operates.

In summary, there is still much to learn about the evolution and ecology of the gut microbiota following antibiotic treatment, but some level of understanding exists. The relative abundances of organisms in the Actinobacteria phylum are often reduced following treatment, while the relative abundances of those in the Bacteroides phylum are often increased, and less clear patterns are shown for the Firmicutes and Proteobacteria phyla (Table 1). The overall composition of the community usually changes during treatment, along with a reduction in diversity, but both factors tend to recover afterwards, although not always and not completely. Antibiotic resistance increases during treatment, which may be caused by extensive horizontal gene transfer, and can persist for years. Meanwhile, the known effects of antibiotics on the mechanisms of evolution suggest that more wide-reaching evolutionary change, that has a range of functional consequences outside of antibiotic resistance alone, is likely.

## PROBLEMS AND SOLUTIONS

Despite many studies that have measured the effects of antibiotic treatment on the gut microbiota, a comprehensive understanding remains elusive. Although differences in methods and reporting make some studies hard to compare, different studies find results that seem to contradict each other. For example, Wipperman *et al*. showed that subjects who had received HRZE antimycobacterial treatment for TB had higher relative *Faecalibacterium* levels than controls (Wipperman *et al*. 2017), while multiple other studies showed that patients had reduced relative *Faecalibacterium* levels during treatment (Dethlefsen *et al*. 2008; Dethlefsen and Relman 2011; Pérez-Cobas *et al*. 2013b). Furthermore, MacPherson *et al*. showed that the total resistance gene abundances in their samples were not significantly increased from the baseline one week after treatment (MacPherson *et al*. 2018), while Jakobsson *et al*. showed that a specific resistance gene remained elevated in their samples four years after treatment (Jakobsson *et al*. 2010).

Even where common conclusions are found between studies, the field is not yet able to fully explain these results or predict, which microbial taxa and genes will be affected. Although it is evident that some species are suppressed due to direct inhibition by antibiotics, we don't know the minimum inhibitory concentrations of many antibiotics for most species of gut bacteria, or how the interactions between different species might contribute to changes in composition and diversity. Similarly, though antibiotic treatment no doubt selects for antibiotic resistance genes, we don't understand the specific mechanisms by which those genes are transferred, what their host ranges are, or in many cases exactly how those genes confer resistance and how they might interact with other aspects of the cell's phenotype and fitness. These gaps in understanding prevent us from being able to predict which subjects would be most at risk for long term microbiota disturbance, or which resistance genes could be expected to persist at high levels after treatment would be an important step towards practical applications of this research. Other microbiome studies have attempted similar things, such as trying to diagnose intestinal disorders based on composition data, but only limited success has been achieved so far (Ryan *et al*. 2020).

**Figure 4.**
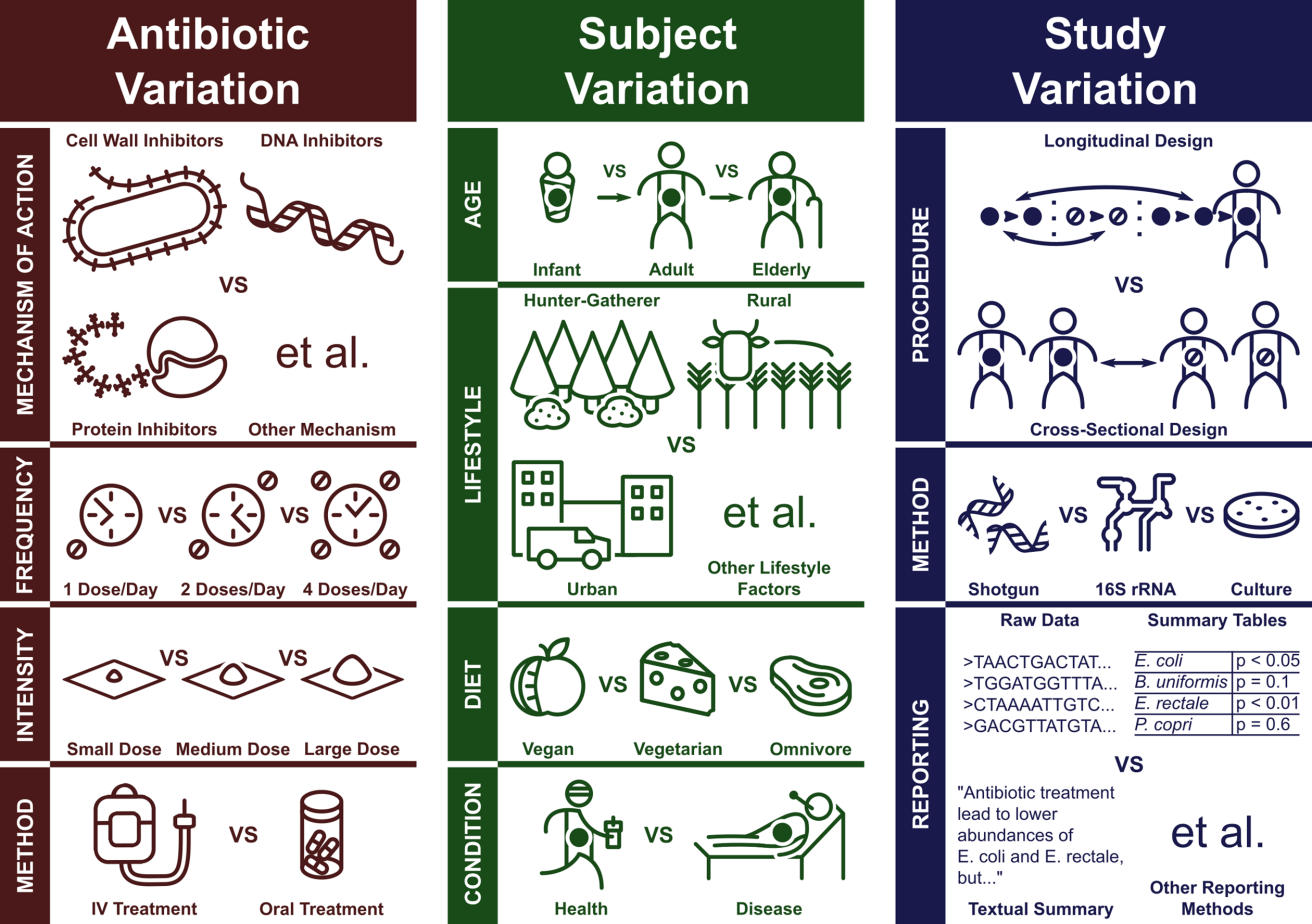
Summary of the main sources of variation that complicate investigation into the effects of antibiotic treatment on the ecology and evolution of the gut microbiome, as expanded upon in the main text.

A major reason why the question, “How does antibiotic treatment affect the gut microbiota?” has yet to find a satisfying answer is that this one question is too broad and serves to mask a multitude of many different, specific, and more guiding questions. For example, “How does a week's course of clindamycin affect the gut microbiota of a healthy Swedish population?”(Jernberg *et al*. 2007) is a very different question to “How does a cocktail of four antibiotics delivered over 6 months affect the gut microbiota of Haitian tuberculosis sufferers?”(Wipperman *et al*. 2017) Variation in the antibiotic treatments used, the subjects receiving treatment, and the specific methods of the study all serve to complicate the question, as summarised in Fig. 4. In the coming sections, we will attempt to outline the full scope of this problem, then suggest how it might be overcome.

### Variation between antibiotic treatments

It is important to make clear that antibiotics are treated as a single group of medicines only because they have similar uses, not because they have similar molecular structures or functions on a biochemical level. Antibiotics can be split into many groups called classes, where each antibiotic class generally contains multiple drugs with variable structures around a common ‘active site’, which has a particular mode of action that interferes with bacterial growth and/or physiology (Aminov 2017). For example, β-lactam antibiotics, like penicillin or amoxicillin, contain an active site which binds to enzymes responsible for building the bacterial cell wall, inhibiting their function and leading to cell death (Cho, Uehara and Bernhardt 2014). Fluoroquinolones, like ciprofloxacin and moxifloxacin, instead bind to enzymes involved in the DNA replication cycle, preventing the cells from generating new DNA (Hooper 2001). Lincosamide antibiotics, like clindamycin, prevent cells from translating mRNA into protein by binding to the bacterial ribosome and interfering with the initiation of peptide chains (Spížek and Řezanka 2017). It seems clear that these completely distinct mechanisms would have different effects on the ecology and evolution of their targets. Some variation based on different classes can be seen in Table 1, such as Arat *et al*.’s study, which used a novel antibiotic treatment with a unique mode of action and found only positive effects on the relative abundance of Actinobacteria and negative effects on the relative abundance of Bacteroidetes (Arat *et al*. 2015). This outcome is in contrast to the general trends we outlined earlier in both directions and suggests that the effects of this novel antibiotic may be atypical.

Beyond the problem of different classes with different mechanisms of action, the intensity, frequency, and duration of antibiotic treatments can vary. Antibiotic courses used in the reviewed studies (Tables 1 and 2) range between one-off doses up to 6-month courses of treatment, involve a single dose per day or up to 4, and vary between 100 mg and 4000 mg doses daily. The relative frequency and intensity by which a community is disrupted can have major impacts on its diversity (Hall *et al*. 2012), so it is reasonable to assume that different doses and treatment schedules may have different effects on the microbiota. Several studies also involved treatments using multiple combined antibiotics, often of different classes, adding a further layer of complexity. It was also found that the effect of antibiotic treatment can depend on whether the drug is administered orally or through an intravenous drip, where in one study it was found that only the oral treatment had a major effect on the gut microbiota (Arat *et al*. 2015).

A study that compared the effects of four different kinds of antibiotic treatment found some distinctly different results between the treatments and concluded that the class of antibiotic plays an important role in modulating the effects on the gut microbiota, as would be expected (Pérez-Cobas *et al*. 2013a). However, with only four total subjects, one receiving each kind of treatment, it is very difficult to separate the effects of different treatments from differences in the individual subjects, or even differences due to random chance. Other studies (Panda *et al*. 2014; Rashid *et al*. 2015; Stewardson *et al*. 2015) also used multiple classes of antibiotics with larger sample sizes. One of these studies found broadly similar effects of beta-lactam and flouroquinolone antibiotics on the gut microbiota, although some effects on specific taxa and total microbial load differed (Panda *et al*. 2014), while another found that ciprofloxacin treatment lead to far greater disturbance of the gut microbiota than nitrofurantoin (Stewardson *et al*. 2015). An *in vitro* study which used faecal culture to thoroughly investigate treatment with seven antibiotics at two different concentrations found distinct differences between their effects (Ladirat *et al*. 2013). These results highlighted how such *in vitro* approaches have the potential to serve as a valuable foundation for further *in vivo* work in this area.

### Variation between treated subjects

The composition of the gut microbiota varies widely between different people. Some research has suggested that human microbiomes can be sorted into several clear ‘enterotypes’ dominated by specific groups of bacteria (Arumugam *et al*. 2011), but the general, recent consensus is that gut microbiota variation is best understood in terms of multiple, continuous gradients rather than a few specific classifications (Knights *et al*. 2014). The most well studied source of gut microbiota variation is diet, where proteins, fats, and carbohydrates—especially indigestible fibre—have characteristic effects on composition (Singh *et al*. 2017). In particular, large differences have been observed between people living traditional hunter-gatherer and rural farming lifestyles compared to those in westernised, urban populations, likely due to a combination of diet and other lifestyle factors (Gupta, Paul and Dutta 2017). The gut microbiota is also known to change as people age, with particularly distinct profiles associated with both extremes of life. Infants begin life with a largely aerotolerant microbiota very different from an adult composition, and later show high levels of *Bifidobacterium* species which are capable of digesting the complex molecules found in human breast milk (Milani *et al*. 2017), while elderly subjects show an overall lower diversity than most adults, with decreased levels of key species such as *Faecalibacterium prausnitzii*, and increased levels of Proteobacteria (Salazar *et al*. 2017). Some studies have even found associations between the sex of subjects and the composition or diversity of the gut microbiota, which may stem from interactions with sex hormones or simply lifestyle differences (Kim *et al*. 2020). Regular exercise seems to have an effect on composition resulting in a greater abundance and diversity of Firmicutes species (Mach and Fuster-Botella 2017). Most importantly, it is clear that many high burden intestinal and extra-intestinal diseases, including inflammatory bowel disease (Kostic, Xavier and Gevers 2014), obesity and diabetes (Patterson *et al*. 2016), and HIV-infection (Bandera *et al*. 2018) are also associated with altered abundances of certain species and groups.

If different people have distinctly different microbiota compositions even before treatment, it is intuitive that these different pre-treatment states will lead to different responses to antibiotics, and there is some clear evidence to support this. For example, in one study, subjects whose microbiota were initially dominated by *Bacteroides* and showed a lower overall diversity were more likely to have increased relative abundance of *Enterobacter cloacae* after treatment (Raymond *et al*. 2016). Stewardson *et al*. showed that a number of subjects that had increased diversity after treatment were dominated by rare genera before treatment (Stewardson *et al*. 2015), and De La Cochetière *et al*. found that the microbial fingerprint of the gut microbiota before treatment was partially able to explain which subjects later acquired *Clostridioides difficile* (De La Cochetière *et al*. 2008). Several studies show other major differences in response between subjects receiving identical treatment, although they were not always able to link these differences to initial conditions, such as certain resistance genes responding in different ways to treatment in Willmann *et al*. (Willmann *et al*. 2015), or different degrees of recovery in Dethlefsen *et al*. (Dethlefsen and Relman 2011).

Subjects in many studies showed distinct individual gut microbiota compositions. For example, variation between subjects remained higher than any variation caused by antibiotics throughout two rounds of treatment in one study (Dethlefsen and Relman 2011). Perhaps more importantly, consistent variation can be observed between the sample groups of different studies. For example, Raymond *et al*.’s subjects were almost all dominated by the Bacteroidetes phylum (Raymond *et al*. 2016), while Jakobsson *et al*.’s and MacPherson *et al*.’s subjects were dominated by the Firmicutes phylum with a lower relative abundance of Bacteroidetes (Jakobsson *et al*. 2010; MacPherson *et al*. 2018). This difference could easily be due to differences in how the samples were collected or how the composition was measured, but it could also reflect true differences in the sample populations used, particularly since Raymond *et al*. used an unusually strict sampling criteria, excluding vegetarians, smokers, and anyone working on a farm or in a healthcare facility. Given that these studies used subject pools with distinctly different initial compositions, and Raymond *et al*.’s study explicitly showed that the initial composition of the microbiota can mediate the effects of antibiotics, the concern arises that any conclusions found might only apply to the specific populations surveyed.

### Variation between research practices

Aside from differences in the treatment and subjects used, further variation can arise from how these studies are carried out and reported. Across various studies that used 16S rRNA sequencing to survey the human gut microbiota, differences in which region of the 16S rRNA gene was targeted and which sequencing methods were used had effects on the data that were sometimes larger than the meaningful biological variables being studied (Lozupone *et al*. 2013). The methods used to identify species from 16S rRNA sequences can also lead to major differences in the final results (Chen *et al*. 2013). Even details such as how much time passes between sample collection and freezing can introduce substantial bias (Cuthbertson *et al*. 2014). In the reviewed studies, it is perhaps most worrying that different results were sometimes found between different methods, even when attempting to measure the same thing. For example, Ladirat *et al*. determined through qPCR that the *Bifidobacterium* genus reduced during treatment, but found no similar effect in their microarray data (Ladirat *et al*. 2014), while O'Sullivan *et al*. found a reduction in bifidobacteria according to selective culture, but not through 16S rRNA pyrosequencing (O'Sullivan *et al*. 2013). These results could simply represent a lack of statistical power in the microarray and pyrosequencing methods, but they still undermine some confidence in any conclusions.

It is clear that in order for results between studies to be meaningfully comparable, a lot of effort needs to be invested in standardising procedures, or at least providing adequate controls to separate the effects of the treatment from various biases. However, out of the studies reviewed, half used no controls at all (Table 2), and others used controls with major differences to their sample group. For example, in one study the untreated subjects were mostly women and all between 26–49 years old, but the treated group was all male and ranged between 1–70 years old (Morotomi *et al*. 2011), and in another all the treated subjects were older than 61 while the controls were younger than 52 (Abeles *et al*. 2015). Many studies also had low numbers of subjects, often in the single digits (Dethlefsen *et al*. 2008; Dethlefsen and Relman 2011; Willmann *et al*. 2015), although others achieved a good balance of treated subjects and controls (Arat *et al*. 2015; Stewardson *et al*. 2015; Raymond *et al*. 2016; Suez *et al*. 2018). Shortcomings in sample size are completely understandable given the difficulty of recruiting volunteers to take antibiotics and donate faecal samples, and in some cases comply with rigid schedules over a large time period, as well as the effort and expense of processing and analysing samples. That said, the lower the sample sizes used in any given study, the more difficult it becomes to correct for these numerous sources of variation.

Even if we assume that 16S rRNA and shotgun metagenomic analyses produce accurate results, there are further problems with how the data is reported. These methods produce vast amounts of data, far more than can be fully comprehended by the researchers or readers. Consequently, researchers typically include only what they consider to be the most important or representative findings in the main text and figures of reports and leave the rest for supplemental data or exclude it entirely. This can lead to problems. A recent review addressed the question of how different antibiotics affect different components of the gut microbiota, and concluded that the genera most sensitive to treatment were *Bifidobacterium, Bacteroides* and *Faecalibacterium* (Ferrer *et al*. 2017). It's not entirely clear how the reviewed papers were summarised, whether it was based on reported changes in the main text, significant associations shown in supplementary data, or re-analysis of raw data, but it is worth noting that these three genera are some of the most well-known and well-studied gut microbiota species. It's entirely possible that these taxa are particularly susceptible to antibiotics, but seems equally likely that findings related to them are reported more often due to their reputation, or that the abundance of reference genomes for these groups makes analyses more powerful and more likely to find significant results, than for lesser-known species.

Problems may also arise from the rapidly shifting taxonomy of bacterial species. *Eubacterium rectale* has recently faced naming disputes, with different parties arguing for its reclassification into *Roseburia, Agathobacter* or a novel genus, although it seems generally agreed that it does not belong within *Eubacterium* (Rosero *et al*. 2016; Sheridan *et al*. 2016; Zuo and Hao 2016). Several other *Eubacterium* species, first isolated and named when classification technologies were far less sophisticated, are being similarly renamed (Rajilić-Stojanović and de Vos 2014). Some reviewed papers mention effects that treatment had on the *Eubacterium* genus (or the Eubacteriaceae family) (Bajaj *et al*. 2013; Pérez-Cobas *et al*. 2013a; Heinsen *et al*. 2015; Stewardson *et al*. 2015; Raymond *et al*. 2016; Wipperman *et al*. 2017), and as time goes on it will be increasingly difficult to interpret which group of organisms this refers to. *Ruminococcus* is another genus with shifting taxonomy (Rajilić-Stojanović and de Vos 2014), and is similarly referred to in some reviewed papers (Dethlefsen and Relman 2011; Morotomi *et al*. 2011; Arat *et al*. 2015; Rashid *et al*. 2015; Stewardson *et al*. 2015; Wipperman *et al*. 2017; MacPherson *et al*. 2018). Without raw sequencing data being made available, which was not the case for several of the reviewed studies (Table S1, Supporting Information), it is impossible for the results of these sorts of studies to remain relevant throughout the constant adjustments to classification and ever improving libraries of reference genomes.

### Suggested future approaches

Considering these problems, how can we make progress towards a comprehensive understanding of how the gut microbiota is affected by antibiotic treatment? We suggest focussing research efforts into two complementary approaches: a top-down, analytic approach which aims to build large, well-annotated databases and to process them with current and future techniques to build predictive models; and a bottom-up, experimental approach which aims to answer specific, mechanistic questions using model systems that afford more replication and control. The key aim is to avoid any middle ground studies which would be too specific to be meaningfully compared to other research, yet too general to answer any questions in detail. Ideally, with these combined approaches, we would be able to move toward both *explaining* and *predicting* the response of the gut microbiome to antibiotic treatment.

Studies contributing to the top-down approach would aspire to recruit large pools of subjects and survey their microbiomes using deep metagenomic sequencing, alongside measurements of metabolites, transcripts, and proteins where possible. The use of untreated controls and several samples taken before treatment would provide multiple meaningful comparisons to determine the effects of antibiotics, while samples taken far after treatment would allow the long-term effects to be measured. Researchers would also collect and report as much metadata as possible, including information related to the subjects’ age, gender, health status, medication, diet, location, etc.; the treatment's dose, frequency, course length, source, etc.; and further details on the collection, processing, and analysis of samples. This approach would result in vast amounts of data, where simplifying results into patterns intuitive to the human mind would be counterproductive, so emerging technologies such as deep learning (Arango-Argoty *et al*. 2018) and other forms of artificial intelligence would be vital to build predictive models which can be validated against further data. While unrelated to antibiotics, a relevant study highlighted the power of this top-down approach; here, researchers were able to predict post-prandial blood glucose levels using a machine learning algorithm which incorporated a broad variety of measurements from 800 subjects, including microbiome composition and function (Zeevi *et al*. 2015). Beyond individual studies, the most crucial aspect of this approach would be effective coordination and collaboration between different groups and disciplines, perhaps lead by funding bodies and/or specific groups that are focused on antibiotic research encouraging researchers to share quality standards, resources, and data (Proctor 2019). No single study could tackle the full breadth of this question, so the success of the top-down approach would depend on practices such as carefully replicating existing studies with different local subject populations, conducting research that makes thorough use of the wealth of existing data rather than producing new information, and making raw data easily accessible. These priorities might result in less studies able to claim individually novel findings, and would certainly require the investment of significant time and resources, but would greatly benefit the research community as a whole.

Studies contributing to the bottom-up approach would draw on the many advantages of conducting research outside of living human guts. *In vitro* fermentation of faecal samples permits experiments with communities approaching the complexity of the natural microbiota, but with far greater capacity for replication and control over their environment, which can lead to much more precise results (Kim *et al*. 2012; Ladirat *et al*. 2013). Animal models, while they must be used with great care, allow the manipulation of host diet, lifestyle and genotype, and for sampling through biopsies and fistulas (Harmoinen *et al*. 2004; Antonopoulos *et al*. 2009), which would be rare or impossible in human studies. Finally, experiments using specific isolates or synthetic communities can allow investigation into basic phenotypic responses to antibiotics (Wassermann *et al*. 2016; Sun *et al*. 2018) or fundamental species interactions (Ze *et al*. 2012). These methods would be used not only to simulate natural perturbations with greater replication and control, but also to formulate specific hypotheses, test them and explain the results in a way that is not currently possible due to the complexity and intractiblity of working with naturally occurring gut microbial communities. For example, Baumgartner *et al*. co-cultured *E. coli* MG1655 with a faecal slurry in the presence of ampicillin and found that despite the presence of horizontally transferable genes in the faecal microbiota, the focal *E. coli* did not evolve resistance (Baumgartner *et al*. 2020). In follow up experiments, they were able to determine that some of these genes could be transferred to the focal isolate, but only on an agar surface rather than in liquid broth, suggesting that the physical environment may have prevented transfer in the full treatment (Baumgartner *et al*. 2020). Another impressive study used a combination of mouse models, human subjects, and culture work to thoroughly investigate the effects of a probiotic on the recovery of the microbiota after treatment and found compelling evidence that secretions of *Lactobacillus* species played a role in delaying recovery (Suez *et al*. 2018). Of course, results like this might not always be applicable to more complex natural systems, but the aim of this approach is not to prove that any individual process or mechanism would certainly occur in the gut, but rather to build a robust library of possibilities that can be consulted to explain natural results.

In time, these two approaches should be able to mutually support each other. Hypotheses generated from narrow, bottom-up experiments could be tested and refined against the large top-down data sets, while emerging patterns from the top-down approach could be explained by the mechanisms demonstrated in the bottom-up approach. An excellent example of this kind of synthesis, within a single paper, comes from a recent study that exploited a large pool of molecular data taken from both novel and previously available cohorts to identify bacterial species suspected to most support microbiota recovery after antibiotic treatment. Then using follow-up experiments with a mouse model the researchers obtained further data that strongly supported their initial results for the two species they had identified and tested (Chng *et al*. 2020). These methods are clearly ambitious, and in many cases will be difficult to achieve due to a variety of time, resource, and organisational constraints. However, as a set of guidelines, these approaches should lead to a far stronger understanding of how antibiotic treatment affects the ecology and evolution of the human gut microbiome in the years to come.

## CONCLUSION

Using the combined approaches outlined above, researchers can work towards both predicting and explaining the response of the human gut microbiota to antibiotic treatment. However, even if the composition of the microbiota could be predicted and manipulated through treatment, we must keep in mind that we still lack a clear understanding of how microbiota composition corresponds to human health (Huttenhower *et al*. 2012; Proctor 2019). More specifically, even if antibiotic mediated effects on community composition and the detailed mechanisms and dynamics of antibiotic resistance evolution could be understood, we still do not know how the presence and absence of different species, and evolved phenotypic traits, are likely to affect disease processes and the transfer of antibiotic resistance to pathogens (van Schaik 2015). Therefore, it is important to acknowledge that without considerable investment into addressing the fundamentals of how microbes influence the human host phenotype, knowledge of the antibiotic mediated effects on microbial ecology and evolution alone might not be immediately medically valuable. However, our understanding of the relationship between the microbiome and host health is constantly progressing, and antibiotic treatment itself represents a valuable investigative tool, since it is known to produce measurable effects on both microbial composition and host health. It is therefore clear that research into a detailed mechanistic understanding of how antibiotics affect the ecology and evolution of the human gut microbiota is a valuable undertaking, not only to advance our fundamental understanding of microbial communities but also to better understand their role in human health.

## Supplementary Material

fuab018_Supplemental_Files
