# Supplementary material for: Ecological and Evolutionary responses to Antibiotic Treatment in the Human Gut Microbiota
Source: FEMS Microbiol Rev. 2021 Apr 6;45(5):fuab018. doi: 10.1093/femsre/fuab018 (PMC8498795; doi:10.1093/femsre/fuab018)
Supplement: fuab018_Supplemental_Files [file fuab018_supplemental_files.zip › Supplementary_Methods.docx]

**Supplementary Methods**

The studies summarised in Table 1, Table 2, and Supplementary Table 1 were chosen as each measured several components of ecology and/or evolution in the gut microbiota of adult, human subjects, during or shortly after exposure to antibiotic treatment. These studies all made a comparison between measurements associated with antibiotic treatment and measurements taken either before treatment or from untreated subjects, in order to determine the effects of treatment on the microbiota. This review is non-exhaustive, and other studies may exist that would also fit these criteria.

The reviewed studies incorporated a wide variety of measurements, analyses, and methods of reporting data, making objective comparison between studies difficult. Using the approaches outlined below, we attempted to summarise key findings from these studies in a way that best preserved accuracy while allowing for easier comparison. Nonetheless, some level of subjectivity was undoubtedly present in our interpretation of results (particularly the interpretation of ordination plots), and in the granularity with which some results were reported. As far as possible, we aimed to interpret results in the same spirit as the authors of each study. In one case (Rashid *et al.*, 2015), we were unable to access supplementary materials that were referenced in the text, and results have been reported from main text only.

We collected results from each study relating to 4 main areas: (1) effects on the diversity of the microbiota; (2) effects on the community composition of the microbiota; (3) effects on individual taxa within the microbiota; and (4) effects on antibiotic resistance genes or phenotypes within the microbiota. In each of these areas, we also distinguished between short- and long-term effects. Often, individual studies found differing results according to different combinations of measurements, comparisons, or treatments. In these cases, any effect found within any subset of data has been reported in Table 1, while Supplementary Table 1 provides more detail. We also recorded whether effects were considered statistically significant, retaining the same definition of significance as the original authors even where they differed between studies (such as whether or not p-values were corrected for multiple tests). In some cases, where an effect seemed apparent but failed to test significantly, where an effect was only shown for a small portion of subjects, or statistical analysis showed an effect that was not visually apparent, we have described effects as ‘possible’ or ‘slight’.

To summarise effects on diversity, we recorded all metrics that measured the richness or evenness within communities, such as the Shannon index, the Simpson index, the Chao1 richness estimator, or phylogenetic diversity. In one case, the dominance index was considered the inverse of diversity, as it measures the absence of evenness.

To summarise effects on community composition, we focused on methods that compared the composition of multiple communities by calculating the distance or dissimilarity between them, such as Bray-Curtis (Bray & Curtis, 1957) or UniFrac (Lozupone & Knight, 2005), or that summarised the meaningful features of multidimensional datasets such as Principal Component Analysis or Redundancy Analysis. If studies addressed composition without using any of these summary methods, those findings were covered under ‘effects on individual taxa’.

To summarise effects on individual taxa, we collected any reported change in the abundance (or relative abundance) of any taxa in response to treatment, often measured by pyrosequencing, shotgun sequencing, culture, or qPCR. The List of Prokaryotic Names with Standing in Nomenclature (Parte, 2018) (Accessed between May 2020 and February 2021) was used to sort these results into the Actinobacteria, Bacteroidetes, Firmicutes, and Proteobacteria phyla. Taxa belonging to other phyla, those which could not be identified to phyla level, or which were not clearly identified in the text, were sorted into ‘Other Taxa’ in Supplementary Table 1. In some cases, the text made clear that more effects were found than explicitly reported, and we have reported results as ‘≥X taxa’. In other cases, it was unclear whether the same or different taxa were affected between different treatments or comparisons, and results are reported as a range of possible effects.

To summarise effects on antibiotic resistance, we collected any information on the levels of genes that are understood to be related to antibiotic resistance, or any demonstration of phenotypic sensitivity to antibiotics.

To distinguish between short- and long-term effects, we sorted collected samples into those taken ‘during’ treatment and ‘after’ treatment, and made a distinction between comparisons made with each group. The ‘during’ group often included samples taken within a few days of treatment, as shown in Supplementary Table 1, to allow the immediate impacts of the drug to subside. Short-term effects were those found in comparisons between ‘during’ and baseline or controls, and long-term effects were those found in comparisons between ‘after’ and baseline or controls. In some cases, ‘during’ samples were compared to all samples before and after treatment, rather than simply before, and results found were interpreted as short term effects.

Several relevant studies informed the text and were referenced throughout, but for various reasons could not be included in Tables 1 and 2. A study by Perez Cobas *et al.* could not be meaningfully summarised in the same way as the other papers, due to the combined complexity of the multiple treatments, multiple measurements, and multiple samples taken during treatment which captured large fluctuations in several variables (Pérez-Cobas *et al.*, 2013a). A study by De La Cochetière *et al*. focused heavily on the development of *Clostridioides difficile* infections in some subjects and did not meaningfully address any of the variables summarised here (De La Cochetière *et al.*, 2008). Finally, a study by Chng *et al*. incorporated data from new cohorts, but only presented results combined with previously available datasets, so the novel data could not be included summarised in the tables (Chng *et al.*, 2020).

The studies represented in Figures 1, 2, and 3 were chosen primarily for the accessibility of the relevant data, and then to show a variety of examples. Figure 1 was created from community composition data provided by Dethlefsen & Relman and by Chng *et al.*’s taxonomic classification of Raymond *et al.* and Zaura *et al.*’s results (Dethlefsen & Relman, 2011, Zaura *et al.*, 2015, Raymond *et al.*, 2016, Chng *et al.*, 2020), processed to produce a non-metric multidimensional scaling of Bray-Curtis distances between samples using the “vegan” package in the R programming language (Oksanen, 2019, R Core Team, 2020). Figure 2 was created from Shannon Index data provided by Jakobsson et al., community composition data provided by Dethlefsen and Relman, and Chng et al.’s taxonomic classification of Zaura et al.’s results (Jakobsson *et al.*, 2010, Dethlefsen & Relman, 2011, Zaura *et al.*, 2015, Chng *et al.*, 2020), processed to calculate Shannon Index values for each sample also using the “vegan” package (Oksanen, 2019). Figure 3 was created from antibiotic resistance data provided by Jernberg *et al.*, Dethlefsen & Relman, and MacPherson et al (Jernberg *et al.*, 2007, Dethlefsen & Relman, 2011, MacPherson *et al.*, 2018). All figures were created using the “ggplot2” package (Wickham, 2016).
